# Editorial: Personal Health Systems

**DOI:** 10.3389/fmed.2020.591070

**Published:** 2020-10-23

**Authors:** Dipak Kalra, Michael Strübin

**Affiliations:** ^1^The European Institute for Innovation Through Health Data, Gent, Belgium; ^2^MedTechEurope, Brussels, Belgium

**Keywords:** personal health records, personal health systems, personal health monitoring, telehealth, mobile health

People increasingly track and collect data on their health, fitness, wellness, and lifestyle, using wearable or portable connected sensors and devices. Almost universally, those devices come connected with an app that users install on a mobile phone or tablet, and a portal where the data is stored. They are marketed as consumer products and are bought by would-be athletes, the “worried well,” and people in between who want to change and improve their lifestyles and become more active.

Increasingly, medical devices also come connected. For example, people with diabetes tracking their blood sugar levels can now routinely send their data to an app and a portal (often hosted by the device vendor), rather than writing it down. These might be shared with health professionals as part of a shared care or continuous monitoring programme. Personal health systems, whether medical or consumer devices, may be relatively passive tools for collecting data, or may be smart: offering feedback, depicting trends or giving advice, generating alerts to the user or remotely signaling a concern to a monitoring service or care professional.

The worlds of consumer devices and medical devices are merging. There is significant industry investment in portable and wearable sophisticated monitoring technologies, apps that are intuitive to use and deliver valuable feedback to users. Medical device manufacturers are developing or buying portals and apps to build patient and user loyalty. There is investment by healthcare providers and authorities to enable their information systems to receive data collected by personal health systems. Advocates of “big data” hope that this wealth of data can be used for analytics, medical evidence, population health management, and precision medicine. The European Health Data Space, announced in late 2019, will advance this vision.

However, there are many challenges to realizing a richer connected world in which patients and healthy citizens co-operate in information, knowledge and decision sharing with health professionals. These include:

- worries about privacy protection and the misuse of personally collected data;
- concerns amongst professionals about the quality of patient generated data and their legal accountability as they devolve more monitoring and care decisions to patients;
- the limited availability of evidence of healthcare value (including cost savings) through the smarter use of personal health solutions;
- the challenges in changing professional culture toward a more equal collaboration and empowered patients and families;
- the slowness of healthcare providers and health authorities to build systems that process patient generated data; and
- the lack of interoperability and the persistence of data silos, with many vendors reluctant to facilitate data sharing with health providers by adopting interoperability standards.

Cumulatively, these barriers have prevented a “break out” of personal connected health: only in few parts of the world have we seen innovative ideas and initiatives taken to scale. It is recognized that the field has fostered many pilots that have failed to be sustained or to scale.

In this Research Topic we offer four papers that elaborate on this analysis of personal health systems, deployed and evaluated implementations, and initiatives that are scaling up.

In “Challenges and Solutions for Designing and Managing pHealth Ecosystems” Blobel emphasizes the importance of holistically considering the human, organizational and technical aspects of personal health systems. The ecosystem in which PHS are deployed and must deliver benefits requires the medication of many collaborating health-related actors. Each actor uses their own specialized domain knowledge that has to be carefully combined within an ecosystem. Blobel advocates that an architectural formalization based on international standards should be used as an over-arching framework on which PHS solutions should be designed.

D'Antrassi et al. have designed, implemented, and evaluated a system for collecting data from patients with Parkinson's Disease within clinical research studies. It can be configured to capture the relevant data for a particular study, which is accessible via a secure cloud to treating clinicians and research investigators. The system was designed to include the data provenance metadata that is required for regulatory submission. This system was evaluated with 10 patients and found high patient acceptance and data accuracy. The approach provides a way for clinical teams caring for patients with Parkinson's Disease to access their clinical research monitoring data.

Lewy et al., in the article “Personalized Health Systems—past, present, and future of research development and implementation in real-life environment,” emphasize the importance of integrating patient generated data and PROMs with EHRs so that clinician-patient decisions benefit from both sources of data. They report on initiatives by Israeli Health Maintenance Organizations demonstrating improved health outcomes and lower costs, accompanied by the introduction of reimbursement policy changes. They conclude that small scale pilots often fail to demonstrate important impacts because of their small size, and that “living pilots” are needed that scale up and become embedded within health systems.

Personally-collected health data needs to be trusted by those whose professional accountability depends on the decisions they make. Patients likewise need to be able to trust that their data is used by people and for purposes that are legitimate. This is especially important when health data is reused for public health and research purposes. Leeming et al. describe the potential for block chain to deliver that capability for trust. In “A Ledger of Me: Personalizing Healthcare Using Blockchain Technology” they present a reference architecture for a blockchain enabled personal health ecosystem and explain the different aspects of trust that the blockchain model could provide.

The field of personal health systems is rapidly expanding to better enable prevention, to improve the accuracy of diagnosis, to scale up self-management, to ensure that clinical decision-making reflects and optimizes patient outcomes, and to enable more personalized medicine. These publications reinforce the evidence that the technologies can now enable us to rapidly expand the engagement of people in collecting and using data themselves, and in partnership with clinicians, and enrich research. However, the time for pilots has passed. To achieve the impact of this potential, personal health system initiatives must include plans for sustainability and scalability from the outset.

## Author Contributions

All authors listed have made a substantial, direct and intellectual contribution to the work, and approved it for publication.

## Conflict of Interest

The authors declare that the research was conducted in the absence of any commercial or financial relationships that could be construed as a potential conflict of interest.

